# Remission of ulcerative colitis flare-up induced by nivolumab

**DOI:** 10.1007/s00384-020-03638-3

**Published:** 2020-05-26

**Authors:** Maho Iwamoto, Kimitoshi Kato, Mitsuhiko Moriyama, Kenya Yamaguchi, Satoru Takahashi

**Affiliations:** 1 Division of Gastroenterology and Hepatology, Department of Internal Medicine, Nihon University School of Medicine, 30-1 Oyaguchi-Kamicho, Itabashi-ku, Tokyo, 173-8610 Japan; 2 Division of Research Planning and Development, Nihon University School of Medicine, 30-1 Oyaguchi-Kamicho, Itabashi-ku, Tokyo, 173-8610 Japan; 3 Division of Urology, Department of Urology, Nihon University School of Medicine, 30-1 Oyaguchi-Kamicho, Itabashi-ku, Tokyo, 173-8610 Japan

**Keywords:** Nivolumab, Immune checkpoint inhibitors (ICIs), Ulcerative colitis (UC), Inflammatory bowel disease (IBD), 5-Aminosalicylic acid (5-ASA)

## Abstract

**Background:**

Immune checkpoint inhibitors (ICIs) have been used to treat many cancers, but ICIs are rarely administered for malignant tumours coexisting with inflammatory bowel disease.

**Methods and results:**

We report a 77-year-old man experiencing an ulcerative colitis (UC) flare-up after receiving nivolumab as third-line therapy for multiple metastases of renal cell carcinoma. Mild UC (proctitis form) had been diagnosed at age 59 years and remission was maintained for 17 years with only a low dose of 5-ASA. After nivolumab treatment, the patient developed diarrhoea, bloody stools and was hospitalised. Computed tomography revealed inflammation involving the entire colon and endoscopy revealed severe UC exacerbation. Histological analysis showed UC findings and also increased crypt apoptosis which is unusual for inflammatory bowel diseases, while being typical of ICI-induced colitis. As with ICI-induced colitis, this exacerbation was strongly suggested to have been caused by nivolumab, although remission was achieved by increasing the 5-ASA dose to 4000 mg without prednisolone.

**Conclusion:**

The administration of ICI for UC is not as yet sufficiently safe and further research is required.

## Introduction

Nivolumab and other immune checkpoint inhibitors (ICIs), which have shown high efficacy against a variety of cancers in recent years, promise long-term survival and even recovery. ICIs are also associated with unique adverse events that are different from those associated with conventional chemotherapy [1, 2]. Immune-related adverse events (irAEs) are attributed to various autoimmune responses that can occasionally become severe and may even be fatal [3–5]. Among them, irAE-associated colitis is reported to closely resemble ulcerative colitis (UC) in endoscopic features and treatment responses [6–9]. A recent report confirmed the efficacy of concurrent administration of infliximab and ICIs [10].

Recently, the number of elderly-onset UC patients has been rising [11]. In elderly patients, the proportion of comorbidities including malignancy unrelated to inflammatory bowel disease (IBD) is high [12]. For these reasons, the number of IBD patients with a comorbid malignancy requiring ICI treatment is expected to increase. However, patients with autoimmune diseases such as IBD have historically been excluded from clinical trials of ICIs, and there are few reports of programmed cell death protein-1 (PD-1) inhibitors administered to patients with a pre-existing form of IBD [13, 14]. Herein, we report an elderly patient with remission of a worsening UC flare-up after nivolumab administration.

## Case presentation

**Fig. 1:**
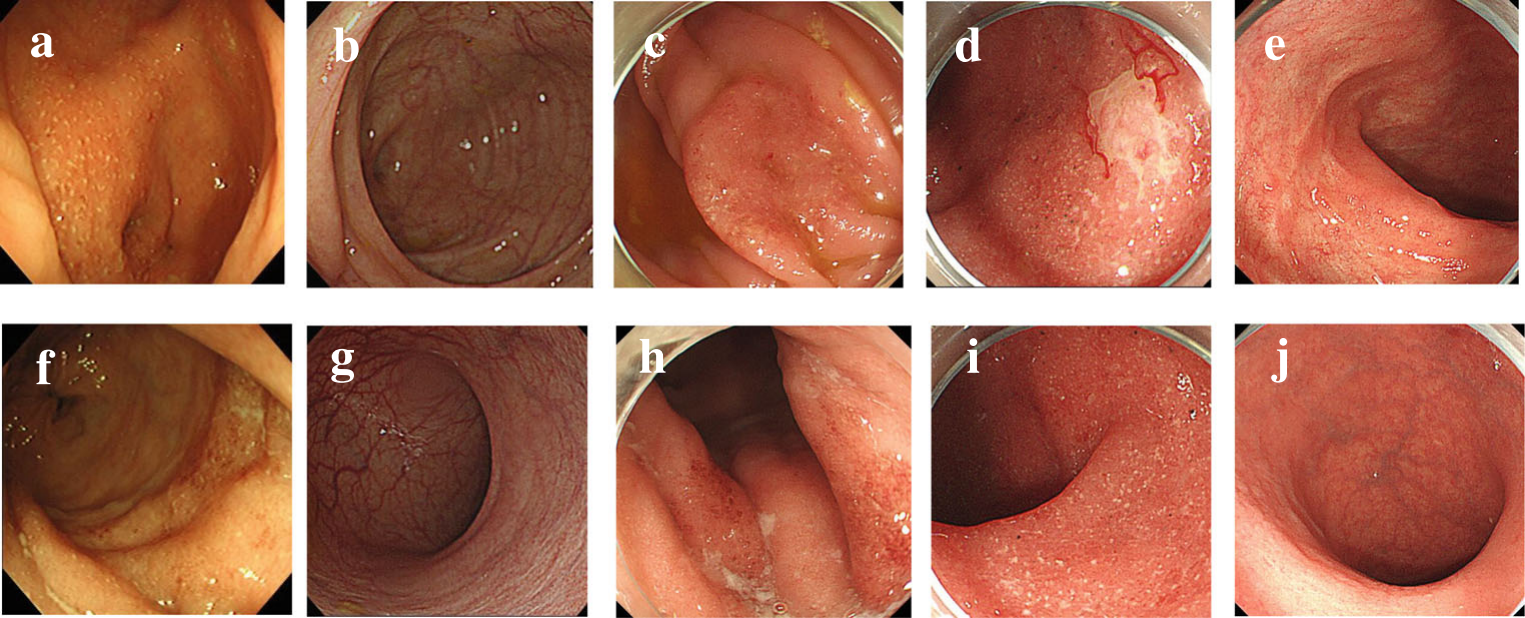
Colonoscopic findings from onset to after discharge. **a**–**e** Sigmoid colon to appendix. **f**–**j** Rectum. **a**, **f** Age 59 years (at onset). **b**, **g** Age 68 years (during maintenance of remission). **c**, **h** Age 76 years and 9 months (3 months after starting nivolumab). **d**, **i** Age 77 years and 1 month (3 months after restarting nivolumab). **e**, **j** Age 77 years and 3 months (after stopping nivolumab and increasing the dose of 5-ASA)

The patient was a 77-year-old man. At 59 years of age, he developed bloody faeces and was diagnosed with mild UC (proctitis form). UC was maintained at a Mayo score of 0 by treatment with 5-aminosalicylic acid (5-ASA) administered as a suppository and orally (Fig. 1a, b, f, g) [15]. At 60 years, he underwent a partial nephrectomy for right renal cell carcinoma; at 65 years, he underwent a total right nephrectomy for local recurrence. At 70 years, the patient developed lung metastasis. Interferon-α (3 million units twice/week) was administered for 3 years but was stopped after the onset of depression. At 73 years, he developed bone metastasis and underwent radiotherapy that failed to achieve a response, then further progressed to gastric metastasis. At 76 years and 3 months, axitinib (10 mg/day orally) was started as the second-line therapy. The lung and metastatic bone foci shrank, and pleural fluid decreased, but the patient developed severe general malaise, and loss of appetite followed by diarrhoea, and axitinib was thus stopped. At age 76 years and 6 months, we confirmed recovery of the patient’s general health and, after obtaining proper informed consent, started nivolumab (3 mg/kg every 2 weeks) as third-line therapy. After 3 months of nivolumab administration, the patient developed diarrhoea 6 times/day, and total colonoscopy revealed a flare-up of UC with a Mayo endoscopic subscore (MES) of 2, extending to the ascending colon from the rectum (Fig. 1c, h) [15]. Symptoms diminished after a temporary cessation of nivolumab; hence, nivolumab was restarted the following month. After 3 months of nivolumab re-administration, the patient developed diarrhoea 8 times/day and also bloody stools. This UC was given a Mayo score of 9, the diarrhoea was judged to be grade 3 according to the CTCAE ver.5, and the patient was hospitalised [16]. Computed tomography revealed inflammation throughout the colon. Endoscopy performed after hospitalisation revealed a more severe exacerbation than before, with an MES of 3 (Fig. 1d, i).

**Fig. 2:**
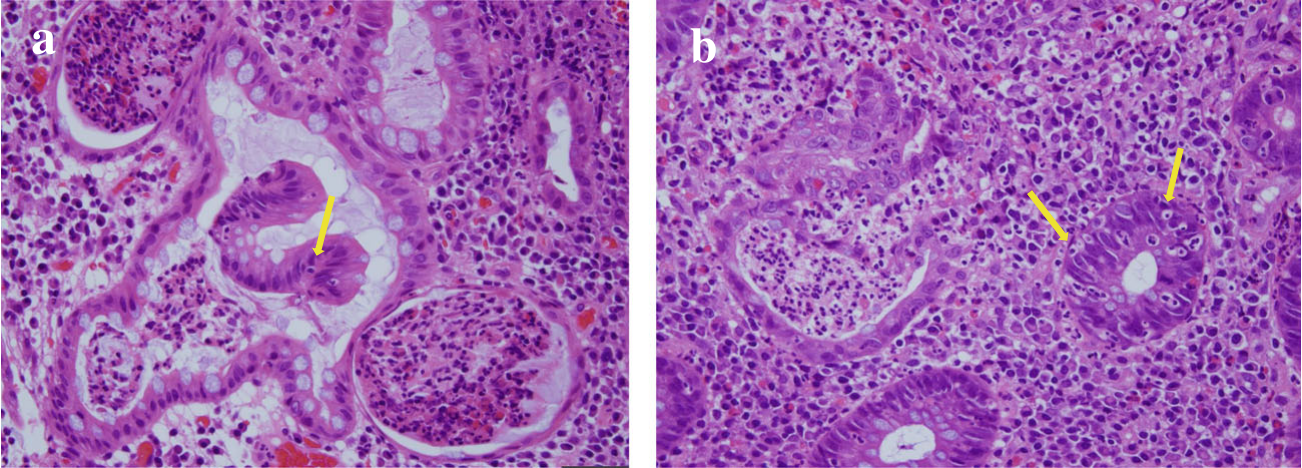
Histological findings after restarting nivolumab included reduced goblet cells, irregular duct layout, cryptitis, crypt abscesses, chronic inflammatory cell infiltrate in the stroma and moderate apoptosis (yellow arrow). Hematoxylin and eosin (H&E) staining × 400. **a** Rectum; **b** sigmoid colon

Histological analysis revealed erosion, reduced goblet cells, irregular duct layout, cryptitis, crypt abscesses and chronic inflammatory cell infiltrate in the stroma. These findings were consistent with UC flare-up, but after recognising increasing apoptosis, we considered the possibility of PD-1 inhibitor-induced enterocolitis to also be high (Fig. 2a, b). Although the diarrhoea was grade 3, we had to consider the advanced age of this UC patient. Hence, we first stopped nivolumab, increased 5-ASA to 4000 mg/day from 1500 mg/day and then observed rapid improvement of symptoms. Without prednisolone administration, the patient was discharged on day 15 of hospitalisation. Following discharge, he continued taking 5-ASA orally at 4000 mg/day with no decrease in renal function and maintained a Mayo score of 0. Another total colonoscopy performed 2 months later revealed continued remission with an MES of 0 (Fig. 1e, j). However, the lung metastasis enlarged, and new liver metastasis was also discovered. We considered restarting nivolumab with concurrent infliximab or vedolizumab, but given the absence of definitive evidence for treating ICI-induced colitis in a patient with UC and because a reduction in tumour size had been achieved with axitinib, we chose to administer low-dose axitinib (5 mg/day orally) as second-line therapy. Although diarrhoea appeared 3 times/day after initiating axitinib administration, the mucosa showed continued remission on endoscopy 6 months later, and this treatment is ongoing.

## Discussion

We have presented a patient with a worsening UC flare-up due to nivolumab treatment. In this patient, the pre-existing UC was of the mild proctitis type, and remission had been maintained for 17 years with only a low dose of 5-ASA. We had difficulty in differentiating this flare-up from ICI-induced colitis; however, as the patient was elderly with pre-existing UC, prednisolone was not administered, and remission was once again achieved by stopping nivolumab and increasing the dose of 5-ASA.

In the few years since the development and approval of ICIs, they have brought about substantial changes in cancer treatment, and we expect that ICI indications will be expanded to include a wide range of malignancies [1, 2]. However, we must consider whether irAEs are becoming a clinical problem [3–5].

Given the mechanism of action of ICIs, there are concerns over an increasing incidence of irAEs and exacerbation of IBD and other autoimmune diseases. This concern has led to very few UC patients being treated with ICIs, and thus very few reports describing such cases.

Including the present case, ICI administration has been confirmed in 10 non-postoperative UC patients. Outcomes after treatment were flare-up in 5 patients (50%) and perforation in one (Table 1) [13, 14, 17–20]. Compared with the reported incidence rates of ICI-induced colitis which range from 0.7 to 22% for ICI monotherapy, the incidence of UC flare-up is high [4, 21].

**Table 1:** Reports on the administration of immune checkpoint inhibitors for non-operated ulcerative colitis patients

Case	Author	Name of ICIs	Age (years)	Sex (M/F)	Type of cancer	Treatment before flare-up	Flare-up	Treatment for flare-up
1	Hijikata et al. [17]	PD-1 (nivolumab)	52	F	Epipharyngeal carcinoma	5-ASA	None	None
2	Kahler et al. [18]	CTLA-4 (ipilimumab)	NS	NS	Melanoma	5-ASA	Colitis (grade 3)	Steroids
3	Kahler et al. [18]	CTLA-4 (ipilimumab)	NS	NS	Melanoma	None	None	None
4	Leonardi et al. [13]	PD-(L)1	NS	NS	Non-small-cell lung carcinoma	Not stated	Non	None
5	Leonardi et al. [13]	PD-(L)1	NS	NS	Non-small-cell lung carcinoma	Not stated	None	None
6	Leonardi et al. [13]	PD-(L)1	NS	NS	Non-small-cell lung carcinoma	Not stated	None	None
7	Bergqvist et al. [19]	CTLA-4 (ipilimumab)	48	F	Melanoma	Vedolizumab	Diarrhoea (grade 3)	Steroids
8	Gutzmer et al. [14]	PD-1 (nivolumab)	51	F	Melanoma	Budesonide, sulfasalazine	Diarrhoea (grade 3)	Steroids
9	Bostwick et al. [20]	CTLA-4 (ipilimumab)	61	M	Melanoma	IFX → AZA	1) Colitis (grade 3) 2) Perforation	1) Steroids IFX → AZA 2) Colectomy
10	Present case	PD-1 (nivolumab)	77	M	Renal cell carcinoma	5-ASA	Diarrhoea (grade 3)	5-ASA dose increase

*ICIs*, immune checkpoint inhibitors; *PD-1*, programmed cell death protein-1; *PD-L1*, programmed death protein ligand-1; *CTLA-4*, cytotoxic T lymphocyte-associated antigen-4; *NS*, not stated; *5-ASA*, 5-aminosalicylic acid; *AZA*, azathioprine; *IFX*, infliximab

Currently, the number of elderly UC patients is increasing globally. Numbers of comorbidities, rates of comorbid infection and perioperative mortality from emergency surgery are also reportedly rising among elderly UC patients, and case fatality rates are correspondingly increasing [11, 22, 23]. As there is likewise an increasing rate of comorbid malignancies unrelated to IBD among elderly UC patients and a rising number of non-operated cases, patients requiring ICI therapy are predicted to increase. Furthermore, when ICIs cause enterocolitis in a UC patient, there is difficulty in strictly differentiating between UC exacerbation and ICI-induced colitis, because the latter often causes endoscopic and histological findings similar to those of UC [6, 7]. In the present case, in addition to the findings of UC, histological analysis showed increased crypt apoptosis which is unusual for IBD, while being typical of ICI-induced colitis [24, 25]. Furthermore, mucosal remission was maintained despite the diarrhoea associated with axitinib. We thus considered nivolumab to be a major contributor to UC relapse.

When enterocolitis occurs after administering drugs to elderly UC patients for comorbid recurrent progressive cancer, rapid therapeutic intervention is first required to prevent UC from flaring up or becoming more severe [11, 12, 22, 23].

Treatment guidelines for ICIs strongly recommend steroids or early administration of infliximab or vedolizumab for ICI-induced colitis. However, this information conflicts with treatment guidelines for elderly IBD patients, for whom steroids or infliximab may increase mortality and the incidence of infection [8, 9, 26, 27]. There is only a single case report of vedolizumab failing to prevent ICI-induced colitis in a UC patient after the administration of a CTCL-4 inhibitor [19].

By contrast, 5-ASA produces a dose-dependent effect, is very safe and is used throughout the world in many IBD patients [28, 29]. Kubo et al. reported improvement of PD-1 inhibitor-induced enterocolitis with only 5-ASA [30]. Our patient also achieved remission with only an increase in the dose of 5-ASA, raising the possibility of 5-ASA being a therapeutic option for flare-ups in UC patients after ICI therapy, especially in patients for whom steroid therapy carries high risk or is even contraindicated.

The administration of ICI for UC is not as yet sufficiently safe and further research is required.

Elderly UC patients who have a recurrent, progressive cancer need flexible treatment strategies that maintain remission of UC and aim for improving the long-term prognosis, which is the primary goal of cancer treatment.
